# Health-Related Quality of Life and Metabolic Control in Children and Adolescents with Type 1 Diabetes Mellitus

**DOI:** 10.4274/jcrpe.2051

**Published:** 2016-03-01

**Authors:** Zeynep Caferoğlu, Neriman İnanç, Nihal Hatipoğlu, Selim Kurtoğlu

**Affiliations:** 1 Erciyes University Faculty of Health Science, Department of Nutrition and Dietetics, Kayseri, Turkey; 2 Nuh Naci Yazgan University Faculty of Health Science, Department of Nutrition and Dietetics, Kayseri, Turkey; 3 Erciyes University Faculty of Medicine, Department of Pediatrics, Division of Pediatric Endocrinology, Kayseri, Turkey

**Keywords:** child, adolescent, type 1 diabetes mellitus, Quality of life, Nutrition

## Abstract

**Objective:**

The burdens imposed on a child and his/her parents by a diagnosis of type 1 diabetes mellitus (T1DM) adversely affect their health-related quality of life (HRQoL). HRQoL is important for prognosis and is related to metabolic control. To evaluate the HRQoL of Turkish children and adolescents with T1DM and to assess the correlation of HRQoL subscales (including physical and psychosocial health) with metabolic control, and particularly with hypo- and hyperglycaemic episodes.

**Methods:**

This cross-sectional study included 70 participants with T1DM aged between 8 and 18 years (study group) and 72 healthy controls who were matched to the study group in terms of age, gender, and sociodemographic characteristics (control group), and their parents. HRQoL was determined by the Pediatric Quality of Life Inventory. As an indicator of metabolic control, the most recent hemoglobin A1c (HbA1c) levels were obtained and the number of hypo- and hyperglycaemic episodes over the past one month were checked.

**Results:**

The study group had similar HRQoL scores for children’s self-reports and parents’ proxy-reports to the control group apart from a decreasing psychosocial health score for parents’ proxy-reports in the study group. Although HbA1c level was not related to HRQoL scores, lower number of hypo- and hyperglycaemic episodes were associated with an increase in psychosocial health scores and physical health scores as well as an increase in the total score for parents’ proxy-reports.

**Conclusion:**

Although there was no correlation between metabolic control and HRQoL in children’s self-reports, the improving HRQoL levels in parents’ proxy-reports were associated with good metabolic control.

**WHAT IS ALREADY KNOWN ON THIS TOPIC?**

Health-related quality of life (HRQoL) is important for prognosis and is related to metabolic control of type 1 diabetes mellitus.

**WHAT THIS STUDY ADDS?**

The higher scores of HRQoL subscales including physical and psychosocial health are associated with metabolic control, particularly hypo- and hyperglycaemic episodes, in Turkish children and adolescents.

## INTRODUCTION

Diabetes mellitus is a group of metabolic diseases characterised by chronic hyperglycaemia resulting from defects in insulin secretion, insulin action, or both (1,2). Type 1 diabetes mellitus (T1DM) continues to be the main type of diabetes encountered in children and adolescents. Over 85% of all diabetes cases in individuals aged <20 years worldwide are T1DM (3). Being diagnosed with T1DM permanently changes the life of children and adolescents. Treatment has some requirements such as frequent insulin injections, daily blood glucose monitoring, diet plan, and regular physical activity. Also, acute and chronic complications related to diabetes may occur. All of these factors can adversely affect the health-related quality of life (HRQoL) in children and adolescents with T1DM (4).

HRQoL is a multidimensional concept including well-being in terms of patient’s physical, emotional, mental, and social behaviours and is defined as the way the effects of a disease and/or its treatment are perceived by the patient (5,6). Well-being can be described in different forms by individuals, and the disease process may also be experienced differently. When evaluating quality of life, it should be considered that there are objective and subjective areas of HRQoL. Two people in the same situation objectively may have different perceptions of their HRQoL subjectively (7). Some researchers suggest that subjective assessment is more valuable because it reflects self-perception about the situation of individuals. However, other researchers indicate that parental forms are more relevant because of objective consequences (7,8,9). Therefore, the evaluation of HRQoL perceived by parents as well as by the children is important to understand the children’s and adolescents’ HRQoL correctly.

The aims of this study were to evaluate the HRQoL of Turkish children and adolescents with T1DM as perceived by both child and parent, and to assess the correlation of HRQoL subscales (including physical health and psychosocial health) with metabolic control, and particularly with hypo- and hyperglycaemic episodes.

## METHODS

This cross-sectional study was conducted between September 2012 and February 2013 on patients attending the Pediatric Endocrinology Outpatient Clinic at Erciyes University Hospital. The study was performed in accordance with the declaration of Helsinki, after informed oral and written consent was obtained from the parents. It was approved by the University of Erciyes Clinical Research Ethics Board (date: 07.08.2012 and reference number: 2012/479).

Sample size was decided as a distinction between intergroup scale scores ≥5 points, statistical power= 0.80 and fallibility= 0.05. The study group consisted of 72 children and adolescents aged between 8 and 18 years diagnosed with T1DM at least 1 year previously and using multiple daily injection insulin therapy and one parent of each child. Exclusion criteria were mental retardation of a severity that made communication difficult and/or having another chronic disease such as coeliac disease, hypothyroidism, etc. In addition, two children with T1DM were excluded from the study because of missing data. The control group consisted of 72 healthy children and adolescents, who were matched to the study group in terms of age, gender and sociodemographic characteristics, and the parents of these children/adolescents. All subjects in the study and control groups were recruited from primary and high schools.

Body weight and height were measured by an experienced dietician, and body mass index (BMI) was calculated with the “weight (kg)/height (m)2” equation (10). The measurements were made with the participants in their underwear, without shoes, standing erect, and placing their head in the Frankfurt plane. An automatic height gauge scale (DENSİ GL150, İstanbul) (11) sensitive to 10-200 kg±50 g and 90-200 cm±1 mm was used for the measurements.

Demographic data including age, gender, socioeconomic status, parents’ education and job, breast feeding duration, eating habits, and physical activity level were collected via a face-to-face interview and recorded on a pre-prepared form. Physical activity level was assessed by the following questions: “Do you perform a physical activity regularly?”, “Define the nature, frequency, and duration of this activity”, and “How many times in the past 7 days did you exercise or participate in sports activities which made you sweat and breathe hard for at least 20 minutes?” (12). Data on diabetes-related information such as age at diagnosis, duration of diabetes, total length of stay in hospital due to diabetes, most recent hemoglobin A1c (HbA1c) value, and the number of hypo- and hyperglycaemic episodes over a period of one month were also collected during these interviews.

The HRQoL levels of the study and control groups were determined by the Pediatric Quality of Life InventoryTM (PedsQLTM) 4.0 Generic Core Scale (13). The validity and reliability of the scale have been tested for Turkish children (7,14). Quality of life is evaluated by the PedsQLTM 4.0 Generic Core Scale in four areas: physical function (eight items), emotional function (five items), social function (five items), and school function (five items). However, three scores are derived from this scale: total score and two subscales’ scores including physical health and psychosocial health covering emotional, social, and school function. When calculating scores, each item is given a score from 0 to 100. Subscales or total scores are calculated by adding the score of each item in a section or total and dividing by the total number of items in the section or total. As the score increases, HRQoL improves. The PedsQLTM 4.0 Generic Core Scale also has appropriate forms for children’s self-reports and parents’ proxy-reports.

After being given information about the purpose and content of the study, participants’ demographic characteristics, eating habits, and physical activity status were determined by a researcher using the above-mentioned questionnaire. Children’s and adolescents’ body weight and height were measured. Participants’ HRQoL levels were assessed using the PedsQLTM 4.0 Generic Core Scale (7,14). Various studies have indicated that the assessment of both parent and child should be considered to understand a child’s HRQoL accurately (7,8,9). Therefore, in this study, the PedsQLTM 4.0 Generic Core Scale was applied to one of the parents as well as their offspring.

In the study group, diabetes-related information including the age at diagnosis, duration of diabetes, and total length of stay in hospital due to diabetes were examined. As an indicator of metabolic control, the most recent HbA1c level was obtained from the medical records of the children. These values were classified as <7.5% optimal, 7.5-9.0% suboptimal, and >9.0% high risk (15). Furthermore, self-monitoring of blood glucose was scanned to the number of hypo- and hyperglycaemic episodes over a period of one month (16). Before starting the study, the patients’ glucometer measurements were checked with their simultaneous laboratory results. The patients were asked to measure their fasting blood glucose at least 4 times per day and to record those values. At the end of one month, the patients’ self-records were checked with automatically records from their glucometers. Hypoglycaemic episodes were defined as when blood glucose levels fall below 70 mg/dL and hyperglycaemic episodes as when blood glucose levels rise above 145 mg/dL-without seizures or coma (15).

### Statistical Analysis

Data were evaluated using IBM Statistical Package for the Social Sciences Statistics 21 statistical software package program. Quantitative variables were analysed with the Shapiro-Wilk test for normality. Because the data did not show a normal distribution, two independent group comparisons were performed by Mann-Whitney U test, and more than two independent groups were compared by Kruskal-Wallis analysis. Also, summary statistics were given as the number (n) and percentage (%) for categorical variables, and median and 25th-75th percentile (Q1-Q3) for numeric variables. When quantitative variables were compared with each other, Spearman correlation analysis was used. Categorical variables were compared by the exact method of chi-square test. Values of p<0.05 were considered statistically significant.

## RESULTS

The demographic characteristics of the participants are given in Table 1. The study and control groups were well matched for age, gender, and socioeconomic status. Also, BMI and parents’ education, job and marital status were similar in the two groups (p>0.05). In the study group, while breastfeeding duration (exclusive plus partial) was shorter than in the control group (p=0.020), birth weight, exclusive breastfeeding duration, and starting age for introduction of cow’s milk were similar to the control group (p>0.05). Children and adolescents in the study group consumed a greater number of meals and had breakfast more regularly than those in the control group (p=0.001). Moreover, performance of physical activity regularly, physical activity frequency, and exercise frequency during the past week were more common among children and adolescents in the study group than in the control group (p<0.05). However, physical activity duration was similar in both groups (p>0.05).

### Diabetes-Related Characteristics of Children and Adolescents with Type 1 Diabetes Mellitus

There was no statistically significant difference between girls and boys in terms of diabetes-related characteristics, as shown in Table 2. The median percentage of HbA1c was 7.80% (7.10-9.03), and the median number of hypo- and hyperglycaemic episodes was 2.5 (0.00-5.25) and 38 (22.00-57.25), respectively.

### Quality of Life Assessment

**Figure 1:**
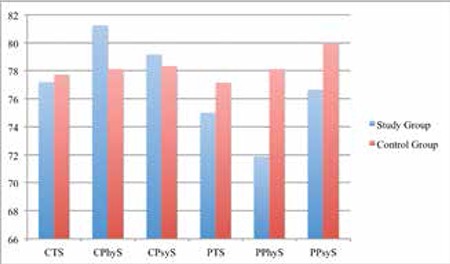
Comparison of groups in terms of health-related quality of life scores. p>0.05 for CTS, CPhyS, CPsyS, PPhyS, PTS and p<0.05 for PPsyS. CTS: Child Total Score, CPhyS: Child Physical health Score, CPsyS: Child Psychosocial Health Score, PTS: Parent Total Score, PPhyS: Parent Physical Health Score, PPsyS: Parent Psychosocial Health Score

In the analysis of the children’s self-reports, no significant differences were found in total scores for HRQoL (p=0.694), for physical health (p=0.359), and psychosocial health (p=0.922) between the study and control groups. However, in the parents’ proxy-reports, the study group parents reported a lower psychosocial health score than the control group parents (p=0.030), while the total scores for HRQoL (p=0.071) and physical health (p=0.269) were similar in both groups (Figure 1).

### The Relationship between Quality of Life and Metabolic Control

In the study group, the correlations between HRQoL scores and metabolic control were evaluated and are shown in Table 3. There was no correlation between HRQoL scores (including total, physical health, and psychosocial health scores) for children’s self-reports and metabolic control. On the other hand, the lower number of hypoglycaemic episodes was associated with increase in psychosocial health scores for parents’ proxy-reports (p=0.031). Also, the lower number of hyperglycaemic episodes was related to increase in the total score of HRQoL (p=0.021) and physical health score (p=0.018) for parents’ proxy-reports. Furthermore, there was no significant difference among the optimal, suboptimal, and high risk groups in terms of HRQoL scores for children’s self-reports and parents’ proxy-reports (p>0.05).

## DISCUSSION

In the treatment of T1DM, intensive therapy programmes are implemented to reduce complications. These intensive therapy programmes place a burden on the children and their family relationships. They usually limit the children’s daily activity, influence the behaviour of the children and their families in a way that is focused on illness and consequently, quality of life may be adversely affected (17). On the other hand, it is known that a diagnosis of T1DM per se creates difficulties for children and adolescents and they usually have difficulties in adapting to the loss of their health and the change in their lives. However, they usually adapt to their disease in the course of time despite their initial perception that their quality of life is impaired (18,19).

In this study, it was shown that Turkish children and adolescents with T1DM have similar HRQoL scores for children’s self-reports and parents’ proxy-reports as healthy controls, apart from a decreasing psychosocial health score noted in the proxy-reports of parents of children with T1DM. The similar perceptions of children with and without T1DM for quality of life are thought to result from the fact that they may have adapted to living with a chronic disease, because of continually living with diabetes for at least a year. However, one individual’s perception of living with a disease may differ from that of another (20). This difference can be demonstrated by looking at the perception of families regarding their children’s status. Unlike the children and adolescents who often have an optimistic view, families tend to indicate that they are adversely influenced by their children having a chronic disease (16,20). These data may explain why HRQoL scores for parents’ proxy-reports in the T1DM group are lower than that of parents of the healthy group, while HRQoL scores for children’s self-reports are similar in the two groups.

The findings of this study are consistent with some previous reports (17,21,22), but are in conflict with others (23,24). Jafari et al (23) showed a statistically significant difference between Iranian children and adolescents with T1DM and healthy controls in terms of HRQoL scores for children’s self-reports and parents’ proxy-reports. In the group with T1DM, these authors reported lower HRQoL scores than in the healthy group. A similar result was found in Kuwaiti children and adolescents (24). These findings have been attributed to be due families not being well informed on the needs of their children, a consequence of the inadequacies in the health system in these communities. Moreover, both studies were conducted at a shorter time after diagnosis as compared to this present study, thus the T1DM patients may not yet have completed the process of adapting to their disease. Thus, the HRQoL scores of children and adolescents with T1DM may differ from healthy controls in some studies.

HRQoL is considered to be an important indicator of prognosis. Children with diabetes experience chronic psychosocial stress and they frequently show more behavioural difficulties and less social competence as compared to healthy children. Therefore, improving HRQoL is important to prevent secondary morbidity and to achieve good metabolic control in the management of diabetes (5,25). In this study, HbA1c level was considered as an indicator of metabolic control. In the majority of studies, the most recently measured HbA1c value was used (16,26,27,28,29,30,31,32,33), although the mean HbA1c during the previous year was also used by some (5,34). In this present study, HbA1c values measured on the day that the questionnaire and PedsQLTM scale were administered were used as an indicator of metabolic control. We found no relationship between HRQoL and HbA1c level, and this finding is consistent with some previous studies (5,26). However, in contrast to this finding, an inverse relationship between HRQoL and HbA1c, decreasing HRQoL scores with increasing HbA1c levels have been reported in many recent studies (27,30,31,33,35,36). At this point, we should note that the lower sample size in our study may have been responsible for our results. Moreover, the fact that the generic HRQoL scale was used to assess the HRQoL of children and adolescents with T1DM in this study, while the diabetes-specific HRQoL scale was used in other studies may also have affected this finding.

Patients with symptoms of hypoglycaemia are more affected by diabetes and they also have more fear and anxiety of hypoglycaemic episodes compared to patients who experience no hypoglycaemia episodes. The potential impact of hypoglycaemia on patients can be explained by considering hypoglycaemic episodes as a barrier to glycaemic control. Increase in the number of hypoglycaemic episodes is associated with reduced glycaemic control, increased cost and also reduced HRQoL (37). The dilemma between tight glycaemic control and risk of hypoglycaemia imposes quite a large burden of disease on young people with diabetes and their families. Treatment and follow-up requirements and the continuous risk of hypoglycaemia (particularly nocturnal hypoglycaemia) adversely affect the HRQoL of patients and their families. As fear of hypoglycaemia increases, the HRQoL of both children and families decreases. Families have a fear of hypoglycaemia which is associated with episodes of severe hypoglycaemia. During severe hypoglycaemia, the child is unconscious or in a coma, not aware of attacks at that moment, and may remember nothing about the incident afterwards. On the other hand, the families witness the incident and hence are perhaps more affected (32).

Hypoglycaemia is a psychosocial barrier as well as a physical barrier for optimal metabolic control and HRQoL. Although some level of fear is a normal response, higher levels are detrimental to HRQoL. Moreover, psychosocial factors often determine self-management behaviours, and psychosocial inconsistencies such as depression are often more powerful predictors of medical outcomes such as hospitalisation and mortality than physical and metabolic measurements such as the presence of complications or high BMI and HbA1c values (32,38). Therefore, the management of T1DM patients should include the evaluation of psychosocial burden including fear of hypoglycaemia imposed by diabetes on children and their families. On the other hand, a reduction in symptoms of hyperglycaemia reported individually is associated with a decrease in diabetes burden and an increase in treatment satisfaction (38). Also, it has been reported that increased HRQoL as perceived by children and their parents, particularly physical health, is related to fewer symptoms of hyperglycaemia (39).

Based on this information, the relationship between number of hypo- and hyperglycaemic episodes and HRQoL subscales including psychosocial and physical health was investigated in this study, and the findings were consistent with those in the literature. It was shown that the increased psychosocial health score for parents’ proxy-reports is related to a decrease in hypoglycaemic episodes, and increased total and physical health scores for parents’ proxy-reports are associated with a reduction in hyperglycaemic episodes. While previous studies reported that total HRQoL score is associated with hypo- and hyperglycaemic episodes (16,40), to the best of our knowledge, this is the first report showing that relationships exist between HRQoL subscales including psychosocial and physical health, especially as perceived by parents, and the number of hypo- and hyperglycaemic episodes.

The findings of this study highlight that poor glycemic control in children and adolescents with T1DM is associated with lower HRQoL scores. These results suggest that it is easier to motivate a child or an adolescent to reach optimal blood glucose levels for improving his/her HRQoL than for preventing long-term diabetes-related complications. In this perspective, routine clinical assessment of HRQoL as an important part of diabetes management may be especially useful in individualizing care and determining the most appropriate interventions.

## Ethics

Ethics Committee Approval: It was approved by the University of Erciyes Clinical Research Ethics Board (date: 07.08.2012 and reference number: 2012/479), Informed Consent: It was taken.

Peer-review: Internal peer-reviewed.

## Figures and Tables

**Table 1:**
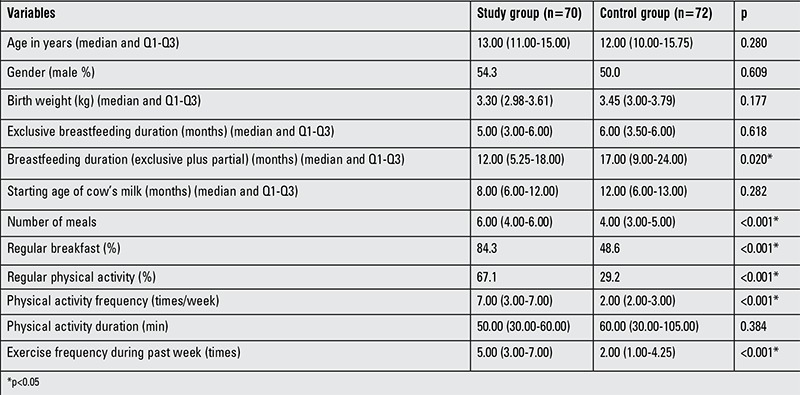
Baseline characteristics

**Table 2:**
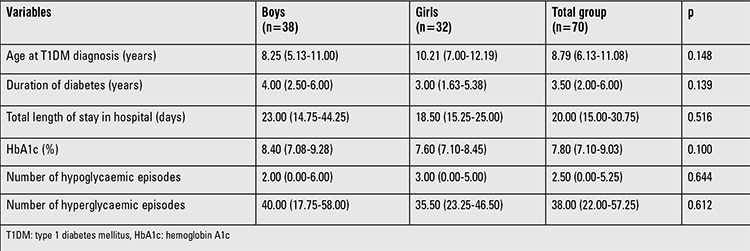
Diabetes-related information in children and adolescents with type 1 diabetes mellitus [median and 25th-75th percentile (Q1-Q3) values]

**Table 3:**
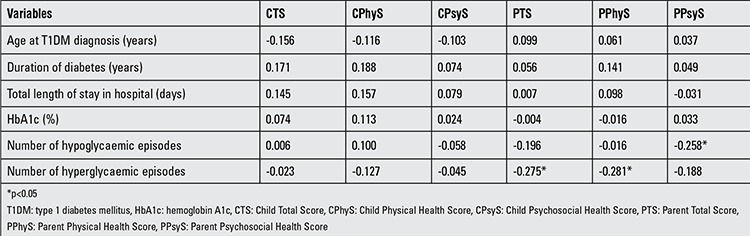
Correlations of health-related quality of life scores with metabolic control indicators and diabetes-related data
